# Immunologic treatment failure among HIV-infected adult patients in Jiangsu province, China

**DOI:** 10.1038/srep42381

**Published:** 2017-02-21

**Authors:** Tao Qiu, Ping Ding, Gengfeng Fu, Xiping Huan, Xiaoqin Xu, Zhi Zhang, Xiaoyan Liu, Haitao Yang, Jeff Mandel, Chongyi Wei, Willi McFarland, Hongjing Yan

**Affiliations:** 1 Institute of HIV/AIDS/STI Prevention and Control, Jiangsu Provincial Center for Diseases Control and Prevention, Nanjing, 210009, China; 2 Jiangsu Institute of Parasite Diseases, Wuxi, China; 3 Global Health Sciences, University of California, San Francisco, California, USA

## Abstract

The National Free Antiretroviral Treatment Program was implemented in Jiangsu Province, China in 2005. We conducted a retrospective, open cohort study to determine treatment failure rates and associated risk factors. Data were obtained from the national web-based antiretroviral treatment database. WHO criteria were used to define immunologic treatment failure. Kaplan-Meier methods were used to determine treatment failure rates and Cox proportional hazards modeling was used to identify risk factors. A total of 5,083 (87.8%) having at least one CD4 cell count measure were included from 2005 to 2013. Overall, 30.4% had immunologic treatment failure with cumulative treatment failure rates increasing to 50.5% at month 60 and 64.1% at month 90. Factors predicting treatment failure included being treated in the Centers for Disease Control and Prevention system (HR 1.69, 95% CI 1.14–2.50, p = 0.009) or jail hospital (HR 1.20, 95% CI 1.08–1.34, p = 0.001), and having a baseline CD4 count >350 cells/uL (HR 2.37, 95% CI 1.94–2.89. p < 0.001). Immunologic treatment failure was moderate to substantial among treated HIV patients. Providing second-line regimens and shifting treatment providers to professional hospitals should be considered to consolidate gains in averting morbidity and mortality.

An estimated 780,000 persons were living with HIV/AIDS (PLWHA) in China as of 2011^1^. In order to reduce HIV-related mortality and new infections, the China National Free Antiretroviral Treatment Program (NFATP) was piloted among former plasma donors in 2002, a population severely affected early in the epidemic, and then scaled up to include other HIV-infected groups^2^,^3^. Antiretroviral therapy (ART) is now more widely available in China. By 2013 the NFATP treated over 209,000 PLWHA and reduced mortality among PLWHA to 14.2 deaths per 100 person years from 39.3 in 2000^4^. Studies have demonstrated the feasibility of providing ART in developing countries, with one-year treatment effectiveness similar to that in developed countries^5^,^6^,^7^,^8^,^9^,^10^,^11^,^12^.

However, after a decade of ART scale up began in earnest, some studies have reported treatment failure and drug resistance among HIV-treated adults in China^13^,^14^. A retrospective cohort study reported a 50% treatment failure rate at five years after treatment initiation among mainly former plasma donors or blood transfusion recipients^11^. A meta analysis showed an overall pooled prevalence of transmitted HIV drug resistance of 3.64% before 2012; however, a significantly higher rate of 5.18% was observed among those initiating ART in the period of 2003 to 2005^15^.

Jiangsu province, located in eastern China, has an estimated number of 12,000 PLWHA. The provincial NFATP was initiated in early 2005. NFATP is a centralized system overseen by the Division of Treatment and Care within the National Center for AIDS/STD Control and Prevention^16^. Implementation and management of the treatment programs, including provision of free first-line ART, are responsibilities of local Centers for Disease Control and Prevention (CDC) and government designated hospitals. In Jiangsu province, only five government designated hospitals in five cities are eligible to provide ART and care to PLWHA. The result has been overburdening of the local CDC in providing treatment and care to the majority of PLWHA.

The pattern of the HIV epidemic in Jiangsu was similar to that of the country, where the main transmission routes were blood-related and injecting drug use in the early phase and gradually changing to sexual contact, both heterosexual and through male-male sex, since 2006^14^. Recent studies found that HIV-positive patients infected through sexual transmission had faster progression of disease than other patients^17^,^18^. Thus, in additional to a more resource-strained centralized system of providing treatment, the changing HIV epidemic may also affect the effectiveness of NFATP. It is therefore important to understand the impact of NFATP on clinical outcomes over the last several years to gain insights for patient management and health planning for expanding treatment. However, long-term data are limited for treatment outcomes among HIV-treated patients in China, in particular among more recently affected groups such as men who have sex with men (MSM). Therefore, we conducted this analysis to examine immunologic treatment failure rates and associated risk factors among a large cohort of HIV-infected adult patients in Jiangsu province, China from 2005 to 2013.

## Results

A total of 5,788 records of treatment eligible HIV-positive individuals who initiated ART in Jiangsu province were collected from the web-based database between January 1, 2005 and December 31, 2013. Of these, 705 (12.2%) had no measures of follow-up CD4 counts and were excluded. These included 473 who enrolled in care in 2013 but had not yet had a CD4 count after ART initiative, 190 who died before follow-up, 38 who migrated to other provinces, and four who were lost to follow up for unknown reasons.

The characteristic of the remaining 5,083 (87.8%) patients who had at least one follow-up CD4 count on record are presented in Table 1. Most (86.9%) were enrolled in care after 2010. The median age at ART initiation was 38 years, 82.1% were men, 56.5% were married. The largest proportion (46.5%) was infected through male-to-male sexual contact. The median baseline CD4 count at ART initiation was 189 cells/uL (interquartile range [IQR], 76 to 285).

**Figure 1:**
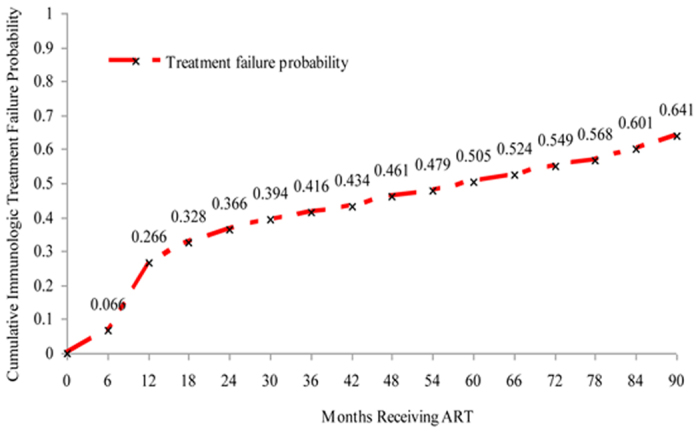
Cumulative treatment failure among patients on antiretroviral treatment (ART) from 2005–2013 (90 months), Kaplan-Meier analysis, Jiangsu province, China.

**Figure 2:**
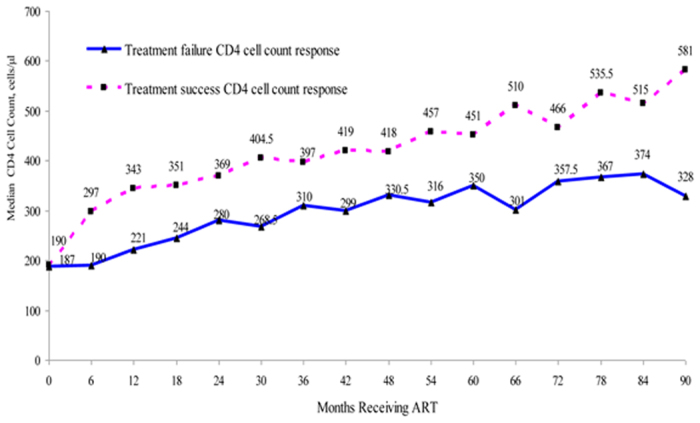
CD4 cell count response among patients by treatment failure vs. success, 2005–2013 (90 months), Kaplan-Meier analysis, Jiangsu province, China.

There were 1,547 patients (30.4%) who met WHO criteria for immunologic treatment failure over the 90-month study period. The cumulative proportion of treatment failure was 26.6% by month 12, 50.5% by month 60, and 64.1% by month 90 (Fig. 1). Differences in the trends in median CD4 cell counts between the treatment success and failure groups are shown in Fig. 2. CD4 cell counts in the treatment success group continued to increase over the 90-month period, whereas CD4 cell counts in the treatment failure group stopped increasing after 60 months.

In bivariate analysis, female sex (HR 0.85, 95% CI 0.75–0.97, p = 0.017), non Jiangsu residency (HR 0.83, 95% CI 0.73–0.95, p = 0.008), and initiating CD4 count 101–200 compared to ≤100 (HR 0.71, 95% CI 061–0.82, p < 0.001) were associated with a lower rate of treatment failure (Table 2). Initiating CD4 count >350 compared to ≤100 (HR 2.29, 95% CI 1.90–2.77, p < 0.001) and treatment at a CDC (HR 1.16, 95% CI 1.05–1.29, p = 0.04) or at a jail (HR 1.92, 95% CI 1.31–2.83, p = 0.001) were associated with higher rates of treatment failure. Of note, age, marital status and mode of transmission were not associated with treatment failure. In multivariate analysis, patients who were treated in the CDC system (HR 1.69, 95% CI 1.14–2.50, p = 0.009) and jail hospital (HR 1.20, 95% CI 1.08–1.34, p = 0.001), and had a baseline CD4 cell count higher than 350 cells/uL (HR 2.37, 95% CI 1.94–2.89, p < 0.001) at ART initiation compared to ≤100, had a higher rate of treatment failure. Compared to ≤100, having a baseline CD4 cell count of 101–200 cells/uL was associated with a lower rate of immunologic treatment failure (HR 0.72, 95% CI 0.62–0.83, p < 0.001).

## Discussion

Almost one-third of patients in Jiangsu province had immunologic treatment failure by one and half years following initiation of ART. Cumulative treatment failure was over half by five years and nearly two-thirds by 7.5 years. The five-year result is consistent with the reported outcome of the national treatment program^12^, which supports that the treatment quality in our province stands the similar level as the nation. However, the accelerated increasing tread of treatment failure in the following 2.5 years alerts Chinese policy makers to evaluate ART effectiveness, second line regimens, and timely monitoring of drug resistance. We also found that CD4 counts continued to improve among patients without treatment failure even to 90 months after initiation; whereas, by 60 months no further improvement was seen among those with treatment failure. Our data suggest that switching to second-line and other drug regimen options will be needed for most patients in Jiangsu province within a few years.

New treatment options will also be needed for many patients earlier in the course of their care. We also found that the cumulative treatment failure rates increased rapidly within the first 12 months of ART initiation. The literature has documented that rates of treatment modification and interruption are high during the first year of ART due to intolerance, toxicity, and depression leading to poor adherence^19^,^20^, which may in turn lead to increased drug resistance. Unfortunately, at present, the national treatment program is limited by few available regimens, limited resources, as well as a lack of a functional drug resistance monitoring system^2^,^3^,^12^. Meanwhile, strategies proven to improve treatment success in the early stages of ART use, such as pre-ART counseling, drug adherence education, and providing social support by peers to reduce stigma, and professional support to treat and reduce depression^21^, should be integrated into existing treatment programs.

We also found patients treated within the CDC system and in jail hospitals had significantly higher rates of treatment failure. This may be due to the lack of HIV medical specialists and fewer clinical resources in these institutions. Other studies in China have shown that patients treated in the CDC systems have higher mortality than those treated in the hospitals^21^. The implications are that China’s national program should significantly increase the number of eligible hospitals to provide treatment for PLWHA, which should engender gains in quality of care, reductions in treatment failure, and subsequent improvements in morbidity and mortality. The timing is especially critical as China’s new national HIV prevention strategy is moving towards scaling up treatment as prevention with more extensive screening of key populations for HIV, identification of undiagnosed HIV cases, and earlier provision of ART^21^.

Puzzling and different from other studies^12^, we found that having a baseline CD4 cell count >350 at ART initiation was a risk factor for treatment failure compared to ≤100. One plausible explanation is tautological in that we used a drop in CD4 cell count to define treatment failure. Individuals with higher baseline CD4 cell counts might be more likely to meet the WHO criterion since their CD4 cell counts were already high and thus even a small drop would categorize them as treatment failure. By a similar rationale, it may be more likely to see an increase in CD4 among patients who had very low baseline counts in response to treatment. We also did not find higher treatment failure rates among patients infected via heterosexual and male-to-male transmission routes as previously reported^17^,^18^. A possible reason could be the shorter observational period among these subgroups as a majority of them were enrolled after 2011.

We recognize other limitations of our study. First, based on the WHO criteria, the definition of immunologic treatment failure did not take into consideration other clinical factors that may affect treatment response. The complicated human immune response mechanism and other possible disease infections might have caused the failure rates to be under- or over- estimated. However, because our study is a long-term cohort with a large sample size, we believe the trends are likely robust. Second, our study did not have patient viral load information and or measures of adherence, also factors measuring or heralding treatment failure^22^,^23^. Finally, there were 12.2% excluded patients who did not have any follow-up CD4 test results, which might have introduced bias to the results of our study. However, 67% of the excluded patients were from 2013, the final year of cohort enrollment, and therefore the bias may be mitigated by the very short observation period compared to the longer term cohort.

Despite limitations, our analysis quantified the moderate to substantial immunologic treatment failure among treated patients in Jiangsu province, China, during a decade of scale up of ART. Providing free or affordable second-line regimens, improving the first-line regimen, including using combinations to increase adherence, and shifting ART providers to professional hospitals should be considered as expansion of treatment continues in order to consolidate the gains in morbidity averted and lives saved so far.

## Methods

### Study Design and Participants

This was a retrospective, open cohort study of HIV-infected adults on ART enrolled between January 2005 and December 2013 in Jiangsu province, China. All patients on ART who had at least one follow-up CD4 count recorded during the observational period were included. Data were obtained from the national web-based ART database of Jiangsu province. The national database has been described in detail elsewhere^3^. Briefly, China’s implementation of the national free ART policy entailed classifying all HIV-positive individuals who meet the updated national treatment guidelines as eligible to receive ART, and they were provided treatment by designated hospitals, CDCs, and jails. The first-line treatment regimen consists of zidovudine (AZT) or stavudine (d4T) or tenofovir (TDF) with nevirapine (NVP) or efavirenz (EFV) and lamivudine (3TC). Except for TDF, all drugs were produced domestically. If patients had treatment failure on the first line regimen, Lopinavir/ritonavir (LPV/r) is used as the second-line regimen drug.

According to the national treatment guideline, after ART initiation clinical follow up visits were scheduled after 2 weeks, 1 month, 2 months, and then every 3 months afterwards. Free CD4 tests were conducted twice per year by designated hospitals or CDC. Baseline CD4 test was required to be conducted within the first 6 months after ART initiation. All clinical visit forms and CD4 results were collected and entered into the national web-based antiretroviral treatment program data information system. The last follow-up clinical visit was up until June 30, 2014.

### Measures

The clinical visit form collected patients’ demographic information, including age, gender, marital status, and registered residence, transmission route, and treatment related information such as facilities providing ART, and CD4 counts (baseline, ART initiation, and follow-ups). We used the WHO criteria to define immunologic treatment failure: CD4 cell count <100 cells/uL after receiving treatment for six months, or CD4 cell count at or less than pretreatment level after receiving treatment for six months, or CD4 cell count less than 50% of peak on-treatment level^24^. We considered treatment to have failed for patients who met any of these criteria. We defined time to failure as treatment initiation date to the first CD4 cell count date when the patient met one of the three criteria. CD4 counts were tested by flow cytometry, FACSCalibur instruments (BD Company, USA). All methods were performed in accordance with the China National Free Antiretroviral Treatment Program, and informed consent was obtained from all subjects in the study.

### Statistical Analysis

Data obtained from the national web-based system were cleaned for analysis. We compared baseline characteristics between treatment success and failure groups by using the Mann–Whitney test for continuous variables and the Pearson chi-square test for dichotomous and categorical variables. We calculated survival curves using the Kaplan-Meier method and assessed statistical significance between groups by using the log-rank test. We used Cox proportional hazards modeling to assess differences in rates of treatment failure as hazard ratios (HR) between potential risk factors. Risk factors with p values < 0.05 in the bivariate COX proportional hazards model were entered into the final multivariate model. SPSS (version 16.0, Chicago, Illinois, USA) was used for all analyses. All statistical testing was 2-sided, with an α level of 0.05.

## Additional Information

**How to cite this article:** Qiu, T. *et al*. Immunologic treatment failure among HIV-infected adult patients in Jiangsu province, China. *Sci. Rep.* **7**, 42381; doi: 10.1038/srep42381 (2017).

**Publisher's note:** Springer Nature remains neutral with regard to jurisdictional claims in published maps and institutional affiliations.

## Figures and Tables

**Table 1:** Patient characteristics at anti-retroviral treatment (ART) initiation, Jiangsu province, China, 2005–2013.

Variable	N (total = 5,083)	Percent
**Year of ART initiation**		
2005–2009	662	13.1
2010	540	10.6
2011	1,053	20.7
2012	1,248	24.6
2013	1,580	31.1
**Guideline on ART eligibility**		
CD4 ≤ 200 cells/uL in period 2005–2009	662	13.0
CD4 ≤ 350 cells/uL in period 2010–2013	4421	87.0
**Age in years**		
≤20	75	1.5
21~40	2,671	52.5
41~60	1,983	39.0
>60	354	7.0
**Sex**		
Male	4,175	82.1
Female	908	17.9
**Marital status**		
Married or cohabiting	2,873	56.5
Single, never married	1,383	27.2
Divorced or widowed	827	16.3
**Jiangsu province residency**		
Yes	4,083	80.3
No	1,000	19.7
**Facility providing ART**		
Hospital	2,360	46.4
Jail hospital	87	1.7
Center for Disease Control and Prevention	2,636	51.9
**Transmission route**		
Blood transfusion	207	4.1
Mother to child	12	0.2
Injecting drug uses	152	3.0
Male to male sex	2,366	46.5
Heterosexual sex	2,319	45.6
Unsure	27	0.5
**Most recent CD4 count prior to ART initiation (cell /uL)**		
≤100	1,536	30.5
101~200	1,109	22.0
201~350	2,078	41.3
>350	313	6.2

**Table 2:** Bivariate and multivariate Cox regression analysis on immunologic treatment failure among patients initiating ART from 2005 to 2013, Jiangsu province, China (N = 5,083).

Variables	Bivariate		Multivariate	
	*P* value	*HR (95%CI)*	*P* value	*HR (95%CI)*
**Guideline on ART eligibility**				
CD4 ≤ 200 cells/uL in period 2005–2009		1		1
CD4 ≤ 350 cells/uL in period 2010–2013	0.008	1.23 (1.06, 1.44)	0.033	1.19 (1.01, 1.40)
**Age**(ys)				
≤20		1		
21~40	0.986	0.10 (0.66, 1.51)		
41~60	0.620	1.11 (0.73, 1.69)		
>60	0.170	1.37 (0.87, 2.14)		
**Gender**				
Male		1		
Female	0.017	0.85 (0.75, 0.97)		
**Jiangsu province residency**				
Yes		1		
No	0.008	0.83 (0.73, 0.95)		
**Marital status**				
Married or cohabiting		1		
Single	0.969	1.00 (0.89, 1.13)		
Divorced or widowed	0.203	1.09 (0.95, 1.25)		
**Facility providing ART**				
Hospital		1		1
CDC	0.004	1.16 (1.05, 1.29)	0.009	1.69 (1.14, 2.50)
Jail hospital	0.001	1.92 (1.31, 2.83)	0.001	1.20 (1.08 1.34)
**Transmission route**				
Blood transfusion		1		
Mother to child	0.702	0.83 (0.31, 2.19)		
Injecting drug uses	0.841	0.92 (0.39, 2.15)		
Male to male sex	0.407	0.79 (0.45, 1.39)		
Heterosexual sex	0.549	0.84 (0.48, 1.48)		
Unsure	0.992	1.01 (0.29, 3.54)		
**Most recent CD4 count prior to ART initiation**				
≤100		1		1
101~200	<0.001	0.71 (0.61, 0.82)	<0.001	0.72 (0.62, 0.83)
201~350	0.255	0.93 (0.83, 1.05)	0.804	0.98 (0.86, 1.12)
>350	<0.001	2.29 (1.90, 2.77)	<0.001	2.37 (1.94, 2.89)

## References

[b1] Ministry of Health, People’s Republic of China Joint United Nations Programme on HIV/AIDS World Health Organization. 2011 Estimates for the HIV/AIDS Epidemic in China. http://www.unaids.org.cn/pics/20130521161757.pdf. Date of access: 09/06/2015 (2011).

[b2] Zhang F. . The Chinese free antiretroviral treatment program: challenges and responses. AIDS. 21 (suppl 8), 143–148 (2007). 10.1097/01.aids.0000304710.10036.2b 18172383

[b3] Zhang F. J., Pan J., Yu L., Wen Y. & Zhao Y. Current progress of China’s free ART program. Cell Res. 15, 877–882 (2005). 16354563 10.1038/sj.cr.7290362

[b4] Zhang F. . Effect of earlier initiation of antiretroviral treatment and increased treatment coverage on HIV-related mortality in China: a national observational cohort study. Lancet Infect Dis. 11, 16–524 (2011). 21600849 10.1016/S1473-3099(11)70097-4

[b5] Stringer J. S. . Rapid scale-up of antiretroviral therapy at primary care sites in Zambia: feasibility and early outcomes. JAMA. 296, 782–93 (2006). 16905784 10.1001/jama.296.7.782

[b6] Severe P. . Antiretroviral therapy in a thousand patients with AIDS in Haiti. N Engl J Med. 353, 2325–2334 (2005). 16319381 10.1056/NEJMoa051908

[b7] Ferradini L. . Scaling up of highly active antiretroviral therapy in a rural district of Malawi: an effectiveness assessment. Lancet. 67, 1335–1342 (2006). 10.1016/S0140-6736(06)68580-2 16631912

[b8] Braitstein P. . Mortality of HIV-1-infected patients in the first year of antiretroviral therapy: comparison between low-income and high-income countries. Lancet. 367, 817–824 (2006). 16530575 10.1016/S0140-6736(06)68337-2

[b9] Ivers L. C., Kendrick D. & Doucette K. Efficacy of antiretroviral therapy programs in resource-poor settings: a meta-analysis of the published literature. Clin Infect Dis. 41, 217–224 (2005). 15983918 10.1086/431199

[b10] Jahn A. . Population-level effect of HIV on adult mortality and early evidence of reversal after introduction of antiretroviral therapy in Malawi. Lancet. 371, 1603–1611 (2008). 18468544 10.1016/S0140-6736(08)60693-5 PMC2387197

[b11] Bussmann H. . Five-year outcomes of initial patients treated in Botswana’s National Antiretroviral Treatment Program. AIDS. 22, 2303–2311 (2008). 18981769 10.1097/QAD.0b013e3283129db0 PMC2853026

[b12] Zhang F. . Five-year outcomes of the China National Free Antiretroviral Treatment Program. Ann Intern Med. 151, 241–251 (2009). 19687491 10.7326/0003-4819-151-4-200908180-00006

[b13] Wu Z., Wang Y., Detels R. & Rotheram-Borus M. J. China AIDS policy implementation: reversing the HIV/AIDS epidemic by 2015. Int J Epidemiol. 39 (Suppl 2), ii1–ii3 (2009). 10.1093/ije/dyq220 PMC2992622 21113031

[b14] Ministry of Health of the People’s Republic of China. 2012 China AIDS Response Progress Report. http://www.unaids.org/sites/default/files/country/documents/ce_CN_Narrative_Report[1].pdf. Date of access: 17/06/2015 (2012).

[b15] Su Y. . The prevalence of HIV-1 drug resistance among antiretroviral treatment naïve individuals in mainland China: a meta-analysis. PLoS One. 9, e110652 (2014). 25343483 10.1371/journal.pone.0110652 PMC4208788

[b16] Dou Z. . Changing baseline characteristics among patients in the China National Free Antiretroviral Treatment Program, 2002–09. Int J Epidemiol. 39, (Suppl 2):ii56–ii64 (2010). 21113038 10.1093/ije/dyq215 PMC2992620

[b17] Li Y. . CRF01_AE subtype is associated with X4 tropism and fast HIV progression in Chinese patients infected through sexual transmission. AIDS. 28, 521–530 (2014). 24472744 10.1097/QAD.0000000000000125

[b18] Yan H. . Emerging disparity in HIV/AIDS disease progression and mortality for men who have sex with men, Jiangsu Province, China. AIDS Behav. 18 (Suppl 1), S5–10 (2014). 23695520 10.1007/s10461-013-0520-2 PMC3864630

[b19] Di Biagio A. . Discontinuation of Initial Antiretroviral Therapy in Clinical Practice: Moving Toward Individualized Therapy. J Acquir Immune Defic Syndr. 71, 263–71 (2016). 26871881 10.1097/QAI.0000000000000849 PMC4770376

[b20] Williams A. B. . Efficacy of an evidence–based ARV adherence intervention in China. AIDS Patient Care STDs. 28, 411–417 (2014). 25046061 10.1089/apc.2014.0070 PMC4117258

[b21] Zhang F. J. . Effect of earlier initiation of antiretroviral treatment and increased treatment coverage on HIV-related mortality in China: a national observational cohort study. Lancet Infect Dis. 11, 516–524 (2011). 21600849 10.1016/S1473-3099(11)70097-4

[b22] Bisson G. P. . Pharmacy refill adherence compared with CD4 count changes for monitoring HIV-infected adults on antiretroviral therapy. PLoS Med. 5, e109 (2008). 18494555 10.1371/journal.pmed.0050109 PMC2386831

[b23] Lima V. D. . The combined effect of modern highly active antiretroviral therapy regimens and adherence on mortality over time. J Acquir Immune Defic Syndr. 50, 529–536 (2009). 19223785 10.1097/QAI.0b013e31819675e9 PMC3606956

[b24] World Health Organization. Antiretroviral therapy for HIV infection in adults and adolescents: recommendations for a public health approach: 2010 revision. Geneva: World Health Organization. http://www.who.int/hiv/pub/arv/adult2010/en/. Date of access:17/06/2015 (2010). 23741771

